# Delayed identification and diagnosis of Huntington’s disease due to psychiatric symptoms

**DOI:** 10.1186/s13033-015-0026-6

**Published:** 2015-08-23

**Authors:** Alina Mihaela Pascu, Petru Ifteni, Andreea Teodorescu, Victoria Burtea, Christoph U. Correll

**Affiliations:** Faculty of Medicine, Transilvania University, Brasov, Romania; Psychiatry and Neurology Hospital, Brasov, Romania; Department of Psychiatry, The Zucker Hillside Hospital, North Shore-Long Island Jewish Health System, Glen Oaks, New York, USA; Hofstra North Shore-LIJ School of Medicine, Hempstead, NY USA

## Abstract

Huntington’s disease (HD) is a progressive neurodegenerative illness that affects 2–9/100.000 of the general population. The usual onset is at around age 35–40 years, but there were cases with onset above 55 years. The disease manifests clinically with many neurological and psychiatric symptoms, leading in advanced phases to dementia, but cognitive symptoms are frequently present much earlier in the disease course. HD is caused by an expanded polyglutamine stretch in the N-terminal part of a 350 kDa protein called huntingtin (HTT). This stretch is encoded by a trinucleotide CAG repetition in exon 1 of HTT. An expansion of greater than 36 repeats results in HD. The number of repeats is inversely correlated with the age of onset of motor symptoms, and disease onset during childhood or adolescence is associated with more than 60 CAG repeats. Mood disturbances may be one of the earliest symptoms of HD and may precede the onset of the motor pheno-type for almost 10 years. Neuropsychiatric symptoms may delay the appropriate diagnosis of HD and have major implications for disease management, prognosis and quality of life for patients and families. This case study is about a 58 years old female patient with late identification of Huntington’s disease after two admissions to psychiatric inpatient units, for the treatment of behavioral disturbances.

## Background

Huntington’s disease (HD) is a progressive neurodegenerative illness, which was the first disorder that was diagnosed using only DNA markers [1]. HD is caused by an expansion of a trinucleotide (CAG) above 35 repeats that is inherited in an autosomal dominant manner, with 100 % penetrance when the CAG expansion exceeds 40 repeats and with incomplete penetrance between 36 and 39 repeats [2]. HD affects 2–9/100.000 of the general population, and has the usual onset at around age 35–40. It manifests clinically by many neurological and psychiatric symptoms, leading in advanced phases to dementia, although noticeable cognitive disturbance is frequently present earlier in the disease [3].

HD is caused by an expanded polyglutamine stretch in the N-terminal part of a 350 kDa protein called huntingtin (HTT). HTT is ubiquitously expressed and is implicated in several cellular functions including control of transcription, vesicular trafficking, ciliogenesis, and mitosis. This stretch is encoded by a trinucleotide CAG repetition in exon 1 of HTT. An expansion of greater than 39 repeats results in HD [4]. The number of repeats is inversely correlated with the age of onset of motor symptoms, and disease onset during childhood or adolescence is associated with more than 60 CAG repeats [5]. Mood disturbances may one of the earliest symptoms of HD and may precede the onset of the motor phenotype for almost 10 years [6–8]. The cognitive symptoms have been shown to be present 15 years prior to the time HD diagnosis and are related to disease-specific cerebral volume loss [9]. Neuropsychiatric symptoms may delay the appropriate diagnosis of HD and have major implications for disease diagnosis, management, prognosis and quality of life for patients and families [10].

This case study is about a female patient diagnosed with HD after two admissions to psychiatric inpatient units, for the treatment of behavioral disturbances. Written informed consent was obtained from the patient for publication of this case report and any accompanying images. A copy of the written consent is available for review by the Editor-in-Chief of this journal.

## Case report

The female patient, 58 years old, was brought to the psychiatric emergency service and hospitalized for psychomotor agitation, verbal and physical aggression towards family, destruction of objects, insomnia, mood lability, dysphoria, and marked irritability. There were no signs of hallucinations, delusions or formal though disorder, the patient denied suicidal ideas. Psychotic or mood disorder diagnoses were excluded. The general physical examination revealed choreiform movements of the upper and lower limbs and head with gait and speech disturbances. The patient’s family declared that these disturbances started 2 years ago, but the patient neglected them. The patient smoked 20 cigarettes/day, but did not use alcohol or any other psychoactive substance. The patient’s father died at the age of 61 after acute renal failure. The mother died at age of 70 after a cerebral stroke. The patient stated that before her mother’s death she presented some abnormal movements of hands and head, but without any neurological examination. The patient’s daughter was advised to perform the genetic test, but she did not follow this recommendation at the time.

Her family reported that 12 months ago the patient had a short episode of loss of consciousness, without seizures, from which she spontaneously recovered and which was not investigated at all. Two years ago, the patient was hospitalized at another psychiatric unit for similar behavioral symptoms considered at that time as an adjustment disorder. The neurological examination did not reveal pathological findings at that time. Thereafter, she was treated as an outpatient for mood symptoms that were considered to be part of a personality disorder.

## Investigations

The routine admission blood work, including electrolytes, renal, hepatic and thyroid parameters as well as full blood count with differential, were all within normal limits. The neurological evaluation found high amplitude choreiform involuntary movements, dysarthria, mild bilateral ataxia, normal sensory and power examination, normal reflexes and mild cognitive impairment (27 points on Mini Mental State Examination). The hospital protocol for psychological evaluation included beyond the MMSE the Raven Standard Progressive matrices for intelligence coefficient (IQ) and Wechsler’s Deterioration Index. The results were normal (IQ = 98 and a deterioration index less than 4 %). The EEG was normal, but the CT scan (without contrast) showed marked, predominantly frontal cortical atrophy, large sylvian valleys and increased size of the ventricular system.

Based on the clinical symptoms, a degenerative illness, such as Huntington’s disease, was suspected. Genetic testing for the HTT gene was performed for confirmation and was analyzed by PCR amplification with specific primers, followed by capillary electrophoresis. For one allele, 43 CAG repeats were detected, and the second allele had 10 CAG repeats. Considering that presence of more than 39 CAG repeats is considered pathological, the conclusion of the genetic analysis was that the patient either already had Huntington’s disease or would develop it in the future.

After the genetic test results had been obtained, the patient was re-evaluated by the neurologist, who established the definite diagnosis of Huntington’s disease, confirmed both on clinical and genetic grounds. She is currently treated with small doses of haloperidol (1 mg/day) and clonazepam (2 mg/day) with good results regarding behavioral and movement symptoms.

## Discussion

This case report is about a patient with late identification of HD at age 58 in a female who was diagnosed for the first time during her second psychiatric hospitalization for psychomotor agitation, aggression, and irritability. The presence of choreiform movements prompted the genetic testing, which together with the clinical picture led to the definite diagnosis of HD, but her diagnosis was delayed by prominence of neuropsychiatric symptomatology.

The particularity of this case is the delayed diagnosis of HD due to psychiatric symptoms and relatively late onset of the illness, based on the history of first emerging abnormal involuntary movements after the age of 55, although the literature usually mentions that the most frequent age of onset is 35–40 years [11].

The onset of the illness was correlated with the beginning of functional impairment, both professionally (she was early retired at age of 56) and socially (she and her husband divorced). The patient was not much aware of or impaired by the abnormal movements and she did not seek medical consultation, as she considered it to be “a nervous tic”. However, the symptom that required medical attention, mostly at her family’s repeated requests, was the behavioral disinhibition. Often, people with HD may show reduced awareness of physical and mental changes in themselves [12]. The lack of awareness was also seen in this patient and is an important corollary of HD that contributes to delays in help-seeking and reporting of newly emerging problems, further delaying the clinical diagnosis and treatment of HD. In our case, although the CT scan showed relevant cortical atrophy, the cognitive impairment was mild, without presence of clinical criteria for a dementia diagnosis. The patient’s psychiatric abnormalities, mainly the behavioral disinhibition, irritability and aggression were likely correlated with the predominantly frontal cortical atrophy. Given the comparatively late onset of HD, the low abnormal number of CAG repeats and mild cognitive dysfunction, it is reasonable to assume that the affected parent in this case was the patient’s mother who had been diagnosed with “cerebral circulatory insufficiency” [13]. Available data suggest that there is a tendency for the affected descendant to present a larger CAG repeat expansion, which is believed to be responsible for a more malignant course of the disease. While gene transmission from the father has been associated with a higher risk for the rigid-akinetic type of HD with childhood onset [14], in women there is an equal risk of contraction or expansion of the CAG repeat [15].

There have been several studies showing that psychiatric symptoms may precede motor symptoms and the ultimate diagnosis of HD [16, 17]. However, in this case, abnormal movements seem to have preceded the psychiatric disturbances by at least 1 year, but it was the latter that led to clinical attention and ultimate diagnosis at least 2 years after the onset of the abnormal movements. This case also illustrates the need to educate psychiatric care providers in recognizing potential neurological reasons for psychiatric presentations, as HD had been missed during the first hospitalization 1 year ago. A suspicion of HD requires the physician to request a genetic test for diagnostic confirmation and, in case of a definite diagnosis, to refer the patient for genetic consultation and counseling, considering that HD is currently incurable and that the descendants of affected parents have a 50 % chance to inherit the disease [18].

## Conclusions

Huntington’s disease is a rare condition which is frequently underdiagnosed, especially early on in the disease. This situation is caused by lack of recognition of HD symptoms, which are often attributed to another disease by patients, family members and sometimes even physicians.
